# Abstract of a Clinical Lecture on the Pathology and Treatment of the Typhoid State in Different Diseases

**Published:** 1868-03

**Authors:** Charles Murchison

**Affiliations:** Physician and Lecturer on the Practice of Medicine at the Middlesex Hospital


### ABSTRACT OF A CLINICAL LECTURE ON THE PATHOLOGY AND TREATMENT OF THE TYPHOID STATE IN DIFFERENT DISEASES.

By CHARLES MURCHISON, M.D., F.R.S.,
Physician and Lecturer on the Practice of Medicine at the Middlesex Hospital.

I propose in the present lecture to direct your attention to several cases of disease, widely different in their nature, but in all of which there has been a similar group of symptoms, constituting the condition ordinarily known as the "typhoid state."

*Case 1.*—William D., aged 23, admitted Oct. 26th, 1866, on the seventh day of an attack of typhus fever, with well-marked eruption; a dry, brown tongue; great delirium and restlessness, and signs of congestion of the lungs. Marked improvement on the fifteenth day. On the eighteenth day, return of the febrile symptoms; an inflammatory swelling over the left parotid, and albumen in the urine. On the nineteenth day, frequent attacks of convulsions, which continued to recur until death by coma on the twentieth day.

*After Death.*—Blood dark and fluid; great hypostatic congestion of lungs; spleen large and soft; kidneys presenting the characters of a recent acute nephritis. A large quantity of clear fluid, containing urea, beneath the arachnoid, and in the lateral ventricles.

*Case 2.*—John F., aged 27, admitted Dec. 3, 1866, on the ninth day of an attack of typhus, with well-marked eruption; a dry, brown tongue, and albumenuria. On the thirteenth day, violent delirium, followed by stupor, floccitatio, and involuntary evacuations, which continued until death on the seventeenth day. No convulsions.

*After Death.*—Blood dark and fluid. Extreme hypostatic congestion of both lungs, the two together weighing sixty-eight ounces. Both kidneys greatly enlarged, weighing together fifteen ounces and a-half, their surfaces smooth, and capsules non-adherent, their cortices extremely congested, and the tubes gorged with granular epithelium. Much clear fluid, in which a considerable quantity of urea was detected, at the base of the brain, and in the lateral ventricles.

*Case 3.*—John S., aged 44, admitted into the London Fever Hospital, on Oct. 12th, 1863, on the seventh day of an attack of typhus, with copious eruption. On the ninth day, acute delirium, followed by stupor, complete unconsciousness, low muttering delirium, and involuntary motions. On the thirteenth day, urine very scanty and smoky, containing epithelial and blood casts. During the following night, a fit of convulsions, lasting a-quarter of an hour, and death by coma half an hour afterwards.

*After Death.*—Blood dark and fluid. Extreme hypostatic congestion of the lungs. Spleen large and soft. The two kidneys together weighing fourteen ounces and a-half, their capsules separating readily, and their cortices quite smooth, but very friable, intensely congested, with blood dripping in large quantity from the cut surface; all the uriniferous tubes gorged with opaque granular epithelium, and many of them containing extravasated blood. Much clear fluid in the lateral ventricles and at the base of the brain, which was not examined for urea.

*Case 4.*—James P., aged 73, admitted Nov. 13th, 1866, with an eruption of pemphigus over the greater part of the body. Many years before he had suffered from "typhus," and from a rheumatic attack, but he had never had dropsy. Two years before, the eruption had appeared on his legs, and within the last three months it had spread over his body. Two or three days after the patient's admission, he became very restless and delirious at night; his tongue was dry and brown, and coarse moist *rales* could be heard over the back of the lungs; the urine was scanty, but though examined repeatedly, contained not a trace of albumen, and there was no dropsy. Death by coma occurred on November 21st.

*After Death.*—The blood dark and fluid. The kidneys contracted and granular, their capsules firmly adherent, and their cortices having well-nigh disappeared. Œdema and hypostatic congestion of the lungs. Much clear fluid, containing urea, at the base of the brain, and in the lateral ventricles.

*Case 5.*—John K., aged 49, admitted Oct. 27, 1866, with a gangrenous ulcer of big toe, the result of an injury, jaundice, and albuminuria. About Nov. 15, the patient became very restless and delirious at night; the tongne was dry and brown; there were signs of hypostatic congestion of the lungs; and the gangrene began to spread up the leg. On Nov. 20th, convulsions occurred, and, after about twenty fits, the patient died comatose on the 21st.

*After Death.*—The cause of the jaundice was ascertained to have been catarrh of the bile-ducts, from irritation of the calculus. Both kidneys were enlarged, and presented the characters of a contracting fatty kidney. Blood dark and fluid. Marked œdema and hypostatic congestion of both lungs. The fluid in the lateral ventricles, and at the base of the brain, was found to contain urea.

*Case 6.*—James McW., aged 42, admitted Oct. 15th, 1867, with symptoms of gangrene of the bladder, succeeding those of the passage of a renal calculus. On Oct. 26th, a rigor, followed by fever, with dry, brown tongue, restlessness, and delirium at night, signs of congestion of the base of the lungs, and death, after an attack of convulsions, on Nov. 7th.

*After Death.*—Gangrene of the bladder; great dilatation of the right ureter. Sacculation of the right kidney, with complete destruction of its secreting tissue; numerous small abscesses in the left kidney. Congestion of the lungs. A considerable quantity of clear serum, containing urea in the lateral ventricles, and at the base of the brain.

*Case 7.*—Henry P., aged 28, admitted Sep. 13, 1866, with all the ordinary symptoms of rheumatic fever in an intense degree, which had lasted about ten days. There was no cardiac complication. On September 17th, he became suddenly worse; he was heavy and stupid, and could scarcely be roused; the urine was loaded with lithates, but contained no albumen; the tongue was dry and brown; there was now faint pericardial friction. On the following day he became violently delirious; the temperature was 107° Fahr., and at 5 P.M., he died comatose. An hour before death, the temperature was found by Dr. Murray to be 110 2° Fahr.; indicating an unusually rapid disintegration, or combustion of tissue.

*After Death.*—Blood dark and fluid. A thin layer of recent lymph over surface of heart, but only half an ounce of fluid in pericardium. Both lungs exhibited in a marked degree the characters of hypostatic congestion, as seen after death from typhus. Spleen ten and a-half ounces, soft and pulpy, like a "typhus-spleen." Both kidneys enlarged and hyperæmic, weighing together twelve ounces and a-half, but surfaces smooth, and capsules separated readily. No fluid in cerebral ventricles.

In this case the products of tissue-metamorphosis, as indicated by the high temperature, were probably in too large quantity for healthy kidneys to eliminate.

*Case 8.*—Esther P., aged 26, admitted Nov. 5th, 1867, with febrile symptoms, a dry, brown tongue and violent delirium. She had been ill for five days before admission, and presented all the symptoms usually observed in a bad case of typhus fever; but, on examination, no eruption could be discovered on the skin, and though there was scarcely any cough, there were all the physical signs of pleuro-pneumonia from base to apex of the left lung, and also at the right base; the urine contained a considerable quantity of albumen. For four days the patient seemed to get worse; she had no sleep, was extremely delirious, and was with difficulty kept in bed; but at the end of that time, an improvement commenced, both in the symptoms and in the physical signs of the lungs; the albumen entirely disappeared from the urine, and on the 6th of December, the patient left the hospital well.

In the cases now submitted to your notice, the group of symptoms known as the "typhoid state" was the same, notwithstanding the varying nature of the primary maladies. These symptoms are a quick, soft pulse; a dry, brown tongue; the symptoms and physical signs of hypostatic congestion of the lungs; impairment of the mental faculties; stupor passing into coma; delirium, which at one time is acute and noisy, at another low and muttering, and not unfrequently associated with muscular tremor; involuntary evacuations, and occasionally subsultus, carphology, or even general convulsions. The precise grouping of the symptoms will vary in different cases even of the same disease, while in diseases essentially different it may be identical.

The *post mortem* appearances met with in such cases are a dark fluid condition of the blood; hypostatic congestion and œdema of the lungs; old disease or recent congestion, with epithelial engorgement of the tubes of the kidneys; enlargement and softening of the spleen, and, unless the typhoid state have been of very short duration, an accumulation of serous fluid in the lateral ventricals and at the base of the brain, the veins and sinuses of which are usually full of dark blood.

The typhoid state is developed more commonly in true typhus fever than in any other disease that we are called upon to treat in this country; and accordingly, all diseases in which the typhoid state is apt to be developed are constantly mistaken for typhus, so that the cases registered in official returns as deaths from "typhus," furnish no reliable test of the prevalence of this epidemic fever. The cases sent as typhus to the London Fever Hospital, and in fact, to all fever hospitals, from year to year, testify unequivocally to the fact that there is scarcely an acute disease to which flesh is heir which may not in the way pointed out be mistaken for typhus. Next to true typhus, enteric, pythogenic, or the so-called "typhoid fever," is the malady in which the typhoid state is oftenest met with; and this is one reason why it was so long, and is still so often, confounded with typhus. In many cases of enteric fever, it is true, the typhoid state does not appear at all, or only late in the disease, and in the latter case the attack is often, though erroneously, described as "gastric fever passing into typhus." Cases of pneumonia are not uncommon where the symptoms of the local disease are completely masked by those of the general blood-poisoning, in the form of manical delirium passing into coma; and there are few medical men who have not met with examples of acute rheumatism passing into a condition indistinguishable from the typhoid state of the typhus, and presenting similar morbid appearances in the solids and fluids after death. Another class of cases very commonly mistaken for typhus is constituted by certain forms of kidney-disease, and particularly the contracted, granular, or "gouty" kidney. In this form of kidney-disease, the patient may at no period of his life have suffered from dropsy, and the urine may contain only a minute trace of albumen; or, as in Case 4, even none at all. The symptoms ordinarily associated with one's ideas of kidney-disease are absent, and, therefore, when cerebral symptoms supervene, the primary disease is overlooked, and the case is regarded as one of typhus; or, if the attack be sudden or ushered in with convulsions, as one of apoplexy. The "serous apoplexy" of the pathologists of the past age—cases diagnosed as apoplexy during life, but where no apoplectic clot was found in the brain after death—were probably, for the most part, examples of contracted kidney. As physician to the London Fever Hospital, numerous cases of contracted kidney have come under my notice during the last six years in which the cerebral symptoms—the typhoid state—were indistinguishable from those of typhus, and where, failing in any previous history, the only points of distinguishing from typhus have been the absence of any eruption on the skin, and the fact that the temperature has been but little elevated above the healthy standard, except when some local inflammation has complicated the primary disease in the kidney.

I shall now endeavor to explain to you why it is that a similar state of the system is so often produced in the course of diseases so widely different, and to show, in fact, that the pathology of the "typhoid state" in all diseases is probably identical, whatever be the cause of the primary disease.

To take, first, the cases of contracted kidney, it is now generally admitted that the cerebral symptoms are due to a poisoned condition of the blood. There may be differences of opinion as to whether the actual poison is urea or some compound of ammonia; but for our present purpose it is immaterial to inquire which of these views is the more probable. It is sufficient to know that the blood is poisoned with the products of the retrograde metamorphosis of the nitrogenous tissues, which it is the function of the kidneys to eliminate from the body. The destruction of tissue is not increased beyond the standard of health, but the disease of the kidneys renders them incapable of separating from the blood the normal quantity of the products of that metamorphosis.

Take next, the class of febrile diseases. It is well known that convulsions—a symptom characteristic of an extreme degree of the typhoid state—is not uncommon in scarlatina, and it has long been acknowledged that this symptom is independent of any cerebral lesion, but is due to poisoning of the blood in consequence of inflammation of the kidneys. Convulsions, however, are met with in many other febrile diseases besides scarlatina, I have known them occur in typhus fever a hundred times or more, and I have published evidence to prove that their pathology in typhus is the same as in scarlet fever. The urine contains always more or less albumen; the kidneys after death exhibit traces of disease, old or recent; and in not a few instances (as in Case 1) I have discovered urea in the fluid of the cerebral ventricles. But convulsions, under such circumstances, are only an extreme symptom of the typhoid state. In a large proportion of cases of typhus, even where no convulsions occur, albumen is present in the urine during the second week of the disease where cerebral symptoms are most apt to appear; and in several cases where death has been preceded for some time by the typhoid state, but not by convulsions, I have found (as in Case 2) urea in the cerebral fluid.

And if this be the pathology of the typhoid state in scarlet fever and typhus, it may fairly be inferred that the typhoid state in all febrile diseases, whether they be due, in the first instance, to a specific poison or to a local inflammation, admits of a similar explanation. As in the contracted kidney, the blood is contaminated with the products of tissue metamorphosis, but the cause of contamination is different. It is now well known that in all febrile disease the disintegration or combustion of the tissue is greatly increased beyond the healthy standard. To this is to be ascribed the increased temperature, which is the pathognomonic symptom of the febrile state, and the rapid emaciation. The products of this increased metamorphosis are mainly eliminated by the kidneys, and appear in the urine. As long as the kidneys are equal to the increased work thrown upon them, the blood is properly depurated, and the typhoid state is warded off. But if the kidneys be unequal to the task, either from the large amount of effete material to be eliminated, from previous disease in the secreting tissue, or from congestion resulting (as it often does) from their increased work, then the blood becomes contaminated, and convulsions or the typhoid state supervene. This explains why albumen so commonly appears in the urine in the course of all acute diseases of a severe form, and why it is so justly regarded as an unfavorable symptom. It also explains why albuminuria is looked upon as a contraindication to any serious surgical operation. The surgical fever which follows an operation is attended by an increased metamorphosis of tissue; and if the kidneys be diseased, the products of the metamorphosis will be retained in the blood, and induce the typhoid state with all its dangers.

If the views now submitted to you as to the pathology of the typhoid state be correct, they furnish material for reflection as to its appropriate treatment. This ought to be directed towards depuration of the blood of the products of tissue metamorphosis, avoiding, at the same time all measures which will irritate the kidneys, and interfere still more with their secreting functions. At the present day, existence of the typhoid state is usually regarded as in itself an indication for liberal administration of alcoholic stimulants. Considerable experience of the typhoid state in all diseases has induced me to doubt the wisdom of the practice, and from an experimental inquiry at the London Fever Hospital, in which the patients admitted with typhus fever on alternate days have been treated with and without alcoholic stimulants, I am satisfied that the benefits of late years ascribed to alcohol in the typhoid state have, to say the least, been greatly overrated. Before drawing conclusions as to the good effects of alcohol in typhus, it is necessary to watch the course of the disease where no alcohol has been given, and this precaution has too often been overlooked. At the same time you must not go away with the impression that stimulants, in moderation, are injurious in all cases where the typhoid state is present. Not unfrequently this condition is accompanied by great impairment of the heart's action, indicated by extreme weakness or irregularity of the radial pulse, a diminution of the cardiac impulse, and weakness with shortening of the first sound. Under such circumstances, stimulants in moderate quantity are unquestionably of use. It is the indiscriminate administration of large quantities of brandy in all cases of the typhoid state that I am induced to condemn, both from theory and from practical experience. Such a practice, it appears to me, is calculated to prove injurious by irritating the kidneys, and thus impeding the elimination of the products of tissue-metamorphosis with which the blood is contaminated. The administration of stimulants must be regulated by the condition of the pulse and heart, and not by the mere existence of the typhoid-state.—*Brit. Med. Jour. Jan. 4, 1868.—Med. News & Library.*

